# Ciliary Defects in Inherited Retinal Diseases

**DOI:** 10.1002/ggn2.202500066

**Published:** 2026-03-06

**Authors:** Guizhi Guo, Lin Li, Jun Zhou, Jie Ran

**Affiliations:** ^1^ Center for Cell Structure and Function College of Life Sciences Shandong Normal University Jinan China; ^2^ Department of Genetics and Cell Biology College of Life Sciences State Key Laboratory of Medicinal Chemical Biology Nankai University Tianjin China

**Keywords:** cilium, gene therapy, inherited retinal disease, photoreceptor, strategy

## Abstract

Inherited retinal diseases (IRDs) are a heterogeneous group of disorders characterized by progressive photoreceptor degeneration that frequently results in severe vision loss. A major cause of IRDs is attributed to structural or functional defects of the photoreceptor cilium that arise from mutations in ciliary genes. The photoreceptor outer segment is a highly specialized sensory cilium composed of hundreds of stacked, flattened, membranous discs. This complex membrane architecture constitutes the primary site of phototransduction, in which light stimuli are converted into biochemical signaling cascades that ultimately generate electrical signals. In this review, the structure and function of photoreceptors are systematically described, major classes of IRDs caused by mutations in ciliary genes are summarized, and the therapeutic potential of emerging ciliary gene‐targeted strategies is critically evaluated in the context of recent advances in IRD treatment.

## Graphical abstract

The photoreceptor cilium is a specialized sensory organelle essential for vision. This review systematically summarizes the structural and functional defects of the cilium that lead to inherited retinal diseases (IRDs). It highlights key pathogenic genes, elucidates molecular mechanisms of degeneration, and evaluates emerging therapeutic strategies targeting ciliary restoration.

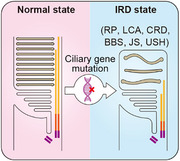

## 1 Introduction

Inherited retinal diseases (IRDs) encompass a diverse group of clinically and genetically heterogeneous disorders that lead to vision loss, with an estimated prevalence ranging from approximately 1:3,000 to 1:4,000 [1]. To date, more than 50 clinical subtypes have been described [2, 3]. Among these, the most prominent subtypes include retinitis pigmentosa (RP), Leber congenital amaurosis (LCA), and cone‐rod dystrophy (CRD) [4]. Clinical manifestations vary substantially across different IRD subtypes and causative genes. RP, the most prevalent subtype, is typically characterized by initial symptoms such as night blindness and progressive constriction of the peripheral visual field. In contrast, LCA is marked by severe visual impairment that manifests at birth or during early infancy [2, 5]. Mutations in approximately 300 genes have been linked to the development of IRDs, with a substantial proportion of these genes encoding ciliary proteins. Consequently, structural abnormalities or functional impairments of the cilium are regarded as a key pathogenic mechanism underlying these disorders [6, 7].

Photoreceptors are highly polarized neurons composed of three distinct subcellular compartments: the outer segment, inner segment, and synaptic terminal. The outer segment, recognized as a specialized primary cilium, serves as the primary site of phototransduction. Its architecture is uniquely adapted for efficient light capture and signal conversion. The photoreceptor cilium is structurally supported by a microtubule‐based axoneme, which extends from the basal body in the inner segment, traverses the connecting cilium (analogous to the transition zone of primary cilia), and continues into the outer segment [8]. Directional transport of molecules, such as opsins, is facilitated by intraflagellar transport (IFT), a mechanism that also supports the continuous renewal of outer segment discs [7, 9, 10]. Therefore, the structural and functional integrity of the photoreceptor cilium is vital for maintaining retinal homeostasis. However, this precise architecture also renders it particularly susceptible to damage under pathological conditions.

Growing evidence links ciliary dysfunction to photoreceptor degeneration in IRDs. Rather than stemming from a single pathway, these disorders frequently arise from defects in a broad array of ciliary processes, including the disruption of axonemal assembly, impairment of IFT machinery, or failure in the specific trafficking of ciliary cargo [7, 8, 11]. Such perturbations inevitably compromise the structural stability of the outer segment and disrupt protein homeostasis, triggering apoptotic cascades that ultimately result in cell death [12]. Given the central role of the cilium in these pathogenic processes, targeting ciliary defects has emerged as a promising therapeutic strategy [11, 13]. In this review, we summarize IRDs associated with ciliary dysfunction, outline emerging ciliary‐targeted strategies aimed at functional restoration, and discuss the potential and challenges of cilia‐centric treatments.

## 2 Morphological and Functional Specializations of the Photoreceptor Cilium

**FIGURE 1:**
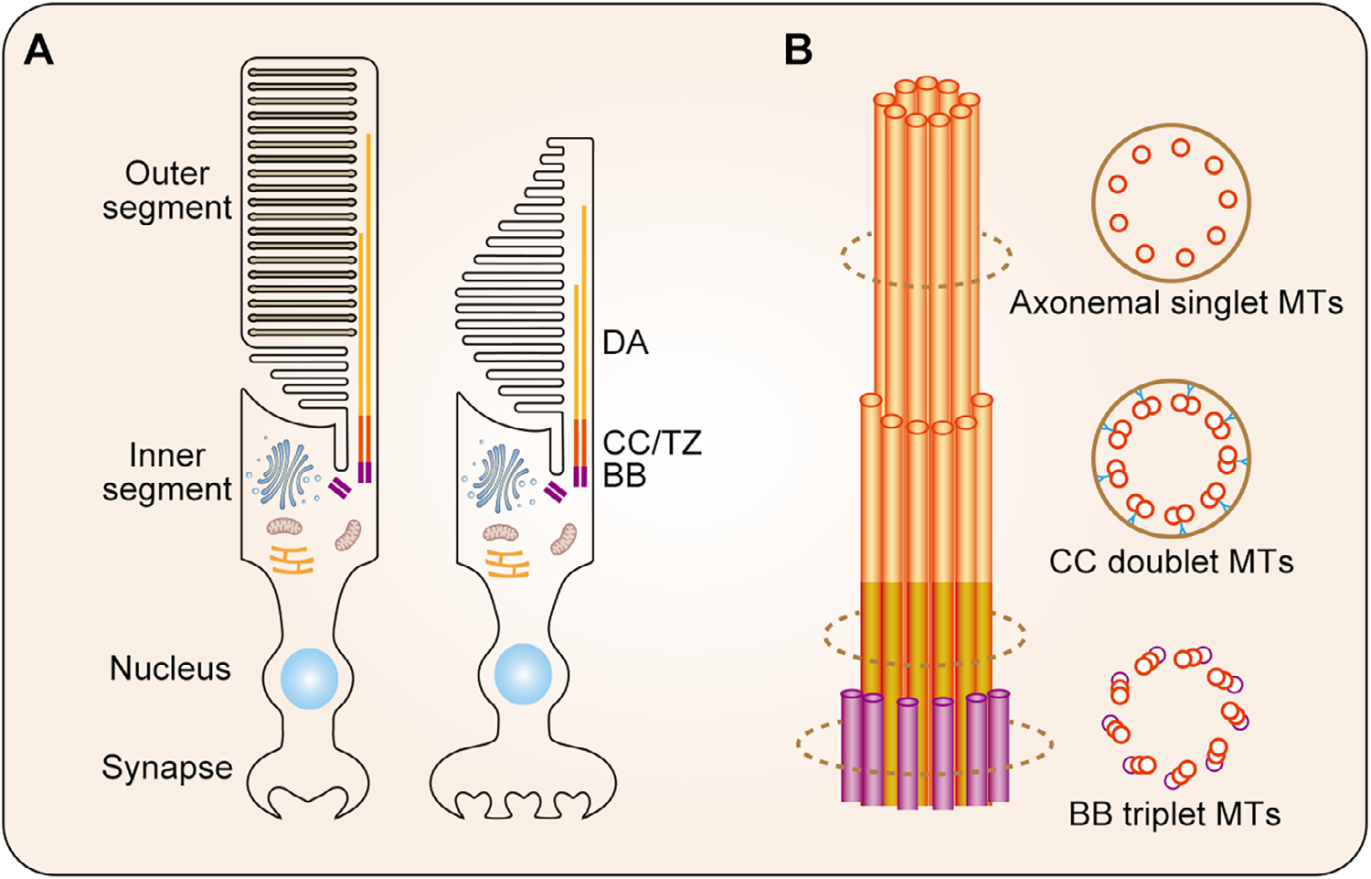
Schematic structure of the photoreceptor. (A) The key compartments of the photoreceptor are the outer segment, inner segment, nucleus, and synapse. The light‐sensing outer segment is connected to the biosynthetic inner segment by the connecting cilium (CC), which facilitates essential intracellular cargo transport. (B) A detailed view of the basal body (BB), CC, and distal axoneme (DA). The BB, composed of triplet microtubules, serves as the microtubule‐organizing center. The microtubule architecture transitions to doublet microtubules in the CC and finally to axonemal singlet microtubules in the distal axoneme, which provide structural support for the outer segment.

Photoreceptor cilium shares a fundamental structural framework with other primary cilia, consisting of an axoneme, a transition zone, and a membranous composition [14]. However, this sensory cilium exhibits unique morphological specializations that distinguish it from most primary cilia. A key feature is the connecting cilium, which bridges the inner and outer segments of the photoreceptor. This region, measuring approximately 1 µm in length, is significantly longer than the typical transition zone of  primary cilium (0.2 µm). Within the distal portion of this region, the axoneme undergoes a characteristic reorganization of its microtubule architecture. The proximal portion retains the canonical “9+0” doublet microtubule arrangement, which gradually transitions to singlet microtubules toward the distal end. Additionally, Y‐shaped linkers anchor the doublet microtubules to the ciliary membrane, forming a stringent diffusion barrier [15]. This barrier selectively regulates the trafficking of proteins and lipids between the inner and outer segments, establishing and maintaining a distinct molecular environment within the outer segment (Figure 1). Such compartmentalization is essential for the specialized function of photoreceptors.

The membrane of the photoreceptor cilium is organized into hundreds of tightly stacked membranous discs, which are critical for light capture and signal transduction. These discs are densely packed at a density of approximately 30 discs per micrometer and are enriched with phototransduction proteins, such as rhodopsin [9]. This organization significantly expands the membrane surface area for photon absorption and enhances signaling efficiency. To sustain this high functional demand, these membranous discs undergo continuous renewal. New discs are generated proximally through membrane evagination, while the oldest distal discs are regularly shed and phagocytosed by the retinal pigment epithelium. This dynamic turnover ensures a constant outer segment integrity, preserving high sensitivity and reliable responsiveness to light [16].

These physiological specializations, while essential for efficient photon capture, impose a unique vulnerability on photoreceptors compared to other ciliated cells. Unlike typical sensory cilia, photoreceptor cilia sustain a massive trafficking load to support the daily renewal of outer segment discs [17]. With ∼10% of discs shed daily, millions of opsin and lipid molecules must traverse the narrow connecting cilium. This bottleneck renders photoreceptors hypersensitive to minor transport defects often tolerated by other cells [9]. Furthermore, the tremendous metabolic load required to maintain the dark current and phototransduction creates a high‐oxygen environment, rendering photoreceptors particularly prone to cumulative oxidative stress [18]. Consequently, this intricate structural and functional integration is highly susceptible to disruption. Even minor defects in disc morphogenesis, protein transport, or photopigment synthesis, which might be tolerated in other cell types, can severely compromise photoreceptor function. Such disruptions contribute to the pathogenesis of various retinal diseases and ultimately lead to vision loss. Thus, a deeper understanding of these specialized ciliary adaptations not only provides critical insights into the mechanisms underlying IRDs but also identifies potential therapeutic targets for clinical intervention.

## 3 Mechanistic Links Between Ciliary Dysfunction and Photoreceptor Degeneration

The photoreceptor cilium is a highly dynamic and specialized organelle, and its structural and functional integrity is maintained by complex molecular machineries. Dysfunction in these processes triggers photoreceptor degeneration through several overlapping pathogenic mechanisms. Understanding these cellular pathways is critical for elucidating the etiology of IRDs. Defects in ciliary genes compromise the structural and functional integrity of photoreceptors through diverse cellular pathways [19]. Here, we categorize these pathogenic mechanisms into four primary domains, ranging from transport defects to secondary stress responses.

### 3.1 Impairment of Intraflagellar Transport and Protein Mislocalization

The biogenesis and continuous renewal of the photoreceptor outer segment necessitate a strictly regulated logistics system, as this organelle is devoid of protein synthetic machinery [20]. Consequently, the IFT apparatus is essential for orchestrating the vectorial translocation of phototransduction components, such as opsins and cyclic nucleotide‐gated channels, from the inner segment through the connecting cilium. This bidirectional trafficking is powered by distinct molecular motors: the anterograde IFT complex (IFT‐B), associated with the heterotrimeric kinesin‐2 motor, drives the delivery of cargoes toward the ciliary tip, whereas the retrograde IFT complex (IFT‐A), driven by the cytoplasmic dynein‐2 motor, mediates the retrieval of turnover products back to the cell body [21].

Dysfunction of this precise transport apparatus inevitably compromises ciliary gating and trafficking fidelity, precipitating the aberrant accumulation of OS‐resident proteins within the inner segment. Such protein mislocalization induces severe cellular stress. For instance, functional loss of *ift88* in zebrafish leads to a complete failure of OS morphogenesis and rapid photoreceptor degeneration driven by the ectopic accumulation of opsins [22]. Furthermore, recent investigations in mouse models have revealed that when IFT‐B is acutely disrupted, photoreceptors attempt to mitigate the resultant proteotoxic stress by sequestering and releasing mislocalized rhodopsin via extracellular vesicles. Despite these compensatory mechanisms, the overwhelming burden of protein mislocalization remains a primary driver of cytotoxicity and cell death, underscoring the indispensability of IFT in preventing neurodegeneration [23].

### 3.2 Compromise of Ciliary Gating and Transition Zone Integrity

The connecting cilium, structurally analogous to the transition zone of primary cilia, functions as a highly selective “molecular gate” that strictly governs the entry and exit of soluble and membrane‐associated proteins. This diffusion barrier is maintained by a sophisticated transition zone protein network—including retinitis pigmentosa GTPase regulator (RPGR), centrosomal protein 290 (CEP290), and NPHP family members, which form Y‐shaped linkers anchoring the axoneme to the ciliary membrane [24]. Disruption of this delicate architecture compromises the barrier's selectivity and structural stability. Recent structural dissections have revealed that this compartment is functionally sub‐compartmentalized. For instance, *SPATA7* has been identified as a critical determinant of the “distal” connecting cilium; its absence precipitates the collapse of axonemal microtubules and the mislocalization of interacting partners like RPGR, thereby destabilizing the entire ciliary conduit [25]. Similarly, the transmembrane protein TMEM138 is indispensable for the precise localization of Abelson helper integration site‐1 (AHI1) to the distal transition zone. Functional loss of *Tmem138* abrogates the vectorial transport of rhodopsin, leading to a complete failure of outer segment morphogenesis and rapid photoreceptor death [26].

Beyond the cilium proper, the integrity of the periciliary membrane base—the docking site for cargo‐bearing vesicles—is equally vital for gating fidelity and transport initiation. Studies in zebrafish demonstrate that the ciliopathy protein CC2D2A orchestrates the assembly of the vesicular fusion machinery (e.g., Syntaxin‐3) at this critical interface. Disruption of this machinery impairs Rab8‐mediated docking, causing the ectopic accumulation of opsins in the inner segment and triggering retinal degeneration [27]. Collectively, these findings underscore that the breakdown of ciliary compartmentalization, whether through structural instability of the transition zone or defects in basal vesicle fusion, disrupts the specialized signaling environment required for vision and serves as a potent driver of pathogenesis.

### 3.3 Aberrant Outer Segment Disc Morphogenesis and Stacking

The continuous renewal of outer segment discs represents a unique morphogenetic feat, necessitating the de novo generation of discrete membranous structures at the ciliary base. This process proceeds through actin‐dependent membrane evagination followed by the organization of the disc rim, a complex structural rearrangement orchestrated by photoreceptor‐specific tetraspanins [9, 28]. Recent molecular dissections have established that PRPH2 and its paralog ROM1 function as the fundamental “building blocks” of this architecture; their assembly into tetrameric complexes provides the structural scaffold strictly required to impose high membrane curvature and define the flattened disc shape [29].

Subsequent to the establishment of the rim scaffold, the nascent discs must undergo a transformative maturation process, termed “disc enclosure”, to physically segregate the disc lumen from the extracellular space [30]. This event is governed by the supramolecular organization of the rim machinery. For instance, investigations in murine models demonstrate that the high‐order oligomerization of PRPH2, beyond the tetramer stage, acts as the specific molecular trigger for disc sealing. Compromise of this oligomerization capability (e.g., via the *Prph2* C150S mutation) abrogates the enclosure process, resulting in the formation of open overgrown membrane lamellae rather than enclosed discs [30, 31]. These structural aberrations preclude the orderly stacking of the outer segment, inducing proteostatic stress and precipitating progressive photoreceptor degeneration.

### 3.4 Induction of Endoplasmic Reticulum Stress and Sterile Inflammation

Beyond structural damage, ciliary defects cause severe stress inside the photoreceptor. When opsins cannot be transported to the outer segment, they pile up in the endoplasmic reticulum (ER) of the inner segment. This traffic jam triggers the Unfolded Protein Response (UPR). While UPR tries to fix the problem at first, unresolved stress eventually forces the cell to commit suicide (apoptosis) [32, 33]. Lin et al. demonstrated that a specific UPR pathway (IRE1 signaling) directly switches the cell from survival to death mode [34]. Furthermore, Gorbatyuk et al. showed that helping the ER fold proteins better (by adding the chaperone BiP) can reduce this stress and save photoreceptors in rat models. This proves that ER stress is a major cause of cell death [35].

In addition to internal stress, the dying retina suffers from chronic inflammation. Damaged photoreceptors release danger signals to the outside, which wake up the immune system. As detailed in recent reviews, this activates resident microglial cells to release inflammatory factors (like IL‐1β). This creates a “vicious cycle”: activated microglia not only clean up dead cells but also mistakenly eat stressed living photoreceptors, making the degeneration much faster than the genetic mutation alone would cause [36].

## 4 Inherited Retinal Diseases Associated With Ciliary Defects

**FIGURE 2:**
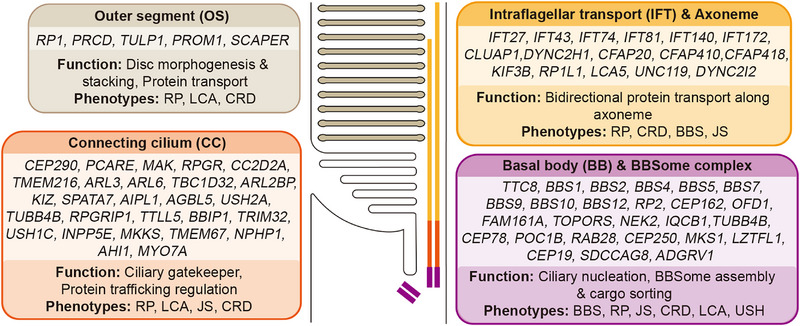
Subcellular localization of protein products and functional classification of ciliary genes associated with inherited retinal diseases. The schematic illustrates the spatial distribution of proteins encoded by representative ciliary genes linked to IRDs across four key compartments: OS, CC, IFT & Axoneme, and BB & BBSome complex. The OS panel includes genes whose protein products are essential for disc morphogenesis and stacking (e.g., *RP1*, *PROM1*). The CC panel highlights genes encoding proteins that regulate the ciliary gatekeeper function and protein trafficking (e.g., *RPGR*, *CEP290*). The IFT & Axoneme panel lists components driving bidirectional transport and structural support (e.g., *IFT* genes, *KIF3B*). The BB & BBSome panel focuses on genes involved in ciliary nucleation and cargo sorting (e.g., *BBS* genes, *OFD1*). For each compartment, the associated biological functions and clinical phenotypes, including retinitis pigmentosa (RP), Leber congenital amaurosis (LCA), cone‐rod dystrophy (CRD), Bardet–Biedl syndrome (BBS), Joubert syndrome (JS), and Usher syndrome (USH), are summarized.

IRDs represent a group of genetically heterogeneous blinding disorders characterized by progressive loss of photoreceptors [37]. Clinically, IRDs can be broadly classified into two categories. Non‐syndromic forms primarily or exclusively affect the retina and include RP, LCA, and CRD. Syndromic forms, on the other hand, occur as components of multisystem disorders, such as Bardet–Biedl syndrome (BBS, associated with retinal degeneration, obesity, polydactyly, and renal abnormalities), Joubert syndrome (JS, characterized by retinal dystrophy, cerebellar malformation), and Usher syndrome (USH, defined by the combination of hearing loss and progressive retinal degeneration) [38]. A substantial proportion of IRD cases result from mutations in ciliary genes, which encode proteins either localized to the cilium or functionally involved in the ciliary signaling pathway [39]. (Table 1 and Figure 2) Accumulating evidence indicates that defects in ciliary assembly, maintenance, and intracellular transport represent central pathogenic mechanisms in IRDs. Such defects disrupt the renewal of the photoreceptor outer segment and compromise its signal transduction capacity [10]. In this section, IRDs mediated by ciliary defects are systematically described.

**TABLE 1:** Representative genes related to IRDs.

Ciliary gene	Phenotype (MIM #)	MIM gene ID	Inheritance	Population genetics	Models
*PCARE* [40]	RP54 (#613428)	613425	AR	Rare	Mouse, Zebrafish, Retinal Organoids
*DYNC2H1* [41]	RP (MIM pending)	603297	AR	Rare	Mouse, Zebrafish
*MAK* [42]	RP62 (#614181)	154235	AR	Founder effect (Ashkenazi Jewish)	Mouse, Zebrafish
*CFAP418* [43, 44]	RP64 (#614500); CRD16 (#614500); BBS21 (#617406)	614477	AR	Rare	Mouse, Zebrafish
*RPGR* [45, 46]	RP3 (#300029); X‐linked CRD (#304020);	312610	XL	Common	Mouse, Retinal Organoids
*KIF3B* [47]	RP89 (#618955)	603754	AD	Rare	Mouse, Zebrafish, Bengal cats
*CC2D2A* [48]	RP93 (#619845); JS9 (#612285)	612013	AR	Rare	Mouse, Zebrafish
*TMEM216* [49, 50]	RP98 (#620996); JS9 (#608091)	613277	AR	Founder effect (Ashkenazi Jewish)	Mouse, Zebrafish
*TTC8* [51, 52]	RP51 (#613464); BBS8 (#608132)	608132	AR	Rare	Mouse, Zebrafish
*ARL6* [53, 54]	RP55 (#613575); BBS3 (#600151)	608845	AR	Rare	Mouse, Zebrafish
*TBC1D32* [55]	RP100 (#621280)	615867	AR	Rare	Mouse, Zebrafish, Xenopus
*ARL2BP* [56]	RP82 (#615434)	615407	AR	Rare	Mouse
*RP1* [57]	RP1 (#180100)	603937	AD, AR	Common	Mouse, Zebrafish
*RP2* [58]	RP2 (#312600)	300757	XL	Rare	Mouse, Zebrafish, Retinal Organoids
*BBS2* [59, 60]	RP74 (#616562); BBS1 (#615981)	606151	AR	Rare	Mouse, Zebrafish
*CEP162* [61]	RP (MIM pending)	610201	AR	Rare	Mouse, Zebrafish
*OFD1* [62, 63]	RP23 (#300424); JS10 (#300804)	300170	XLR	Rare	Mouse, Zebrafish
*ARL3* [64, 65]	RP83 (#618173); JS35 (#618161)	604695	AD, AR	Rare	Mouse, Retinal Organoids
*IFT140* [66]	RP80 (#617781);	614620	AR	Rare	Mouse, Zebrafish, Retinal Organoids
*IFT172* [67]	RP71 (#616394); BBS20 (#619471)	607386	AR	Rare	Mouse, Zebrafish
*IFT43* [68]	RP81 (#617871)	614068	AR	Rare	Mouse
*CFAP20* [69]	RP (MIM pending)	617906	AR	Rare	Mouse, Zebrafish
*CFAP410* [70]	RP (variant)/RD with macular staphyloma (#617547)	603191		Rare	Mouse
*FAM161A* [71]	RP28 (#606068)	613596	AR	Founder effect (Ashkenazi Jewish)	Mouse, Zebrafish
*KIZ* [72]	RP69 (#615780)	615757	AR	Rare	Mouse, Zebrafish
*PRCD* [73]	RP36 (#6100599)	610598	AR	Rare	Mouse, Dog
*SPATA7* [74]	RP94 (#604232); LCA3 (#604232)	609868	AR	Rare	Mouse
*TULP1* [75, 76]	RP14 (#613843); LCA15 (#604232)	602280	AR	Rare	Mouse
*RP1L1* [77]	RP88 (#618826)	608581	AR	Rare	Mouse
*TOPORS* [78]	RP31 (#609923)	609507	AD	Rare	Mouse, Zebrafish
*NEK2* [79]	RP67 (#615565)	604043	AR	Rare	Mouse, Zebrafish
*AGBL5* [80]	RP75 (#617023)	615900	AR	Rare	Mouse, Zebrafish
*AIPL1* [81, 82]	RP (#604393); LCA4 (#604393); CRD (#604393)	604392	AD, AR	Common	Mouse, Retinal Organoids
*PROM1* [83, 84]	RP41 (#612095); CRD12 (#612657)	604365	AD, AR	Rare	Mouse, Retinal Organoids
*SCAPER* [85]	RP (#618195)	611611	AR	Rare	Mouse, Zebrafish
*USH2A* [86, 87]	RP39 (#613809); USH2A (#276901)	608400	AR	Common	Mouse, Zebrafish, Retinal Organoids
*CEP290* [88, 89, 90]	LCA10 (#611755); BBS14 (#615991); JS5 (#610188)	610142	AR	Common	Mouse, Zebrafish, Retinal Organoids
*TUBB4B* [91]	LCA (#617879)	602660	AD	Rare	Mouse, Zebrafish
*IQCB1* [92]	LCA/SLSN5 (#609254)	609237	AR	Rare	Mouse, Zebrafish, Retinal Organoids
*LCA5* [93]	LCA5 (#604537)	611408	AR	Rare	Mouse, Zebrafish, Retinal Organoids
*RPGRIP1* [94, 95]	LCA6 (#613826); CRD13 (#608194)	605446	AR	Rare	Mouse, Zebrafish, Dog, Retinal Organoids
*CLUAP1* [96]	LCA (MIM pending)	616787	AR	Rare	Mouse, Zebrafish
*CEP78* [97]	CRD (#617236)	617110	AR	Rare	Mouse
*POC1B* [98]	CRD20 (#615973)	614784	AR	Rare	Mouse
*IFT81* [99]	CRD (MIM pending)	605489	AR	Rare	Mouse, Zebrafish
*DYNC2I2* [100]	CRD (MIM pending)	613363	AR	Rare	Mouse
*RAB28* [101]	CRD18 (#615374)	612994	AR	Rare	Mouse, Zebrafish
*TTLL5* [102]	CRD19 (#615860)	612268	AR	Rare	Mouse, Zebrafish
*UNC119* [103]	CRD24 (#620342)	604011	AD	Rare	Mouse, Zebrafish
*CEP250* [104, 105]	CRD (#618358); USH (MIM pending)	609689	AR	Rare	Mouse
*BBS9* [106]	BBS9 (#615986)	615986	AR	Rare	Mouse, Zebrafish
*MKS1* [89, 107]	BBS13 (#615990); JS28 (#617121)	609883	AR	Founder effect (Finnish)	Mouse, Zebrafish
*BBS1* [108]	BBS1 (#209900)	209901	AR, DR	Common	Mouse, Zebrafish
*BBS4* [109]	BBS4 (#615982)	600374	AR	Rare	Mouse, Zebrafish
*BBS5* [110]	BBS5 (#615983)	603650	AR	Rare	Mouse, Zebrafish
*BBS7* [111]	BBS7 (#615984)	607590	AR	Rare	Mouse, Zebrafish
*BBS10* [112]	BBS7 (#615987)	610148	AR	Common	Mouse, Zebrafish
*BBS12* [113]	BBS7 (#615989)	610683	AR	Rare	Mouse, Zebrafish
*BBIP1* [114]	BBS18 (#615995)	613605	AR	Rare	Mouse, Zebrafish
*LZTFL1* [115]	BBS17 (#615994)	606568	AR	Rare	Mouse, Zebrafish
*CEP19* [116]	BBS (MIM pending)	615586	AR	Rare	Mouse
*SDCCAG8* [117]	BBS16 (#615993)	613524	AR	Rare	Mouse, Zebrafish
*IFT27* [118]	BBS19 (#615996)	615870	AR	Rare	Mouse, Zebrafish
*IFT74* [119, 120]	BBS22 (#617119); JS40 (#619582)	608040	AR	Rare	Mouse, Zebrafish
*MKKS* [121]	BBS6 (#605231)	604896	AR	Founder effect (Amish)	Mouse, Zebrafish
*TRIM32* [122]	BBS11 (#615988)	602290	AR	Founder effect (Hutterite, Bedouin)	Mouse, Zebrafish
*TMEM67* [89, 107]	BBS14 (#615991); JS6 (#610688)	609884	AR	Rare	Mouse, Rat, Zebrafish
*NPHP1* [123]	JS4 (#609583)	607100	AR	Common	Mouse, Zebrafish
*AHI1* [124]	JS3 (#608629)	608894	AR	Rare	Mouse, Zebrafish
*INPP5E* [125]	JS1 (#213300)	613037	AR	Rare	Mouse, Zebrafish
*MYO7A* [126]	USH1B (#276900)	276903	AR	Common	Mouse, Zebrafish, Retinal Organoids
*ADGRV1* [127]	USH2C (#605472)	602851	AR, DD	Rare	Mouse, Zebrafish
*USH1C* [128]	USH1C (#276904)	605242	AR	Founder effect (Acadian/Cajun)	Mouse, Zebrafish

*Note*: RP, Retinitis Pigmentosa; LCA, Leber Congenital Amaurosis; CRD, Cone‐Rod Dystrophy; BBS, Bardet–Biedl Syndrome; JS, Joubert Syndrome; USH, Usher Syndrome; AD, Autosomal Dominant; AR, Autosomal Recessive; DD, Digenic; XL, X‐linked; XLR: X‐linked Recessive.

### 4.1 Non‐Syndromic Inherited Retinal Diseases

#### 4.1.1 Retinitis Pigmentosa (RP)

RP is a prevalent form of inherited retinal degeneration, characterized by progressive photoreceptor death and consequent severe vision loss. RP exhibits high genetic heterogeneity, and its inheritance patterns primarily include Autosomal Recessive (AR), Autosomal Dominant (AD), and X‐linked Recessive (XLR) modes [129]. The pathology typically begins with the degeneration of rod photoreceptors, leading to night blindness, followed by the loss of cone photoreceptors, which results in central visual impairment [130]. Globally, RP affects approximately 1 in 3,000 to 1 in 7,000 individuals, with an estimated prevalence of 1 in 4,000 in the Chinese population [131].

To date, more than 200 genes have been implicated in RP, including a growing number that encode proteins localized to the photoreceptor cilium [132]. Mutations in these genes disrupt cilium architecture or interfere with ciliary protein trafficking, ultimately leading to photoreceptor degeneration. For instance, the X‐linked gene *RPGR* encodes a protein predominantly localized to the photoreceptor connecting cilium, where it anchors the ciliary gatekeeper network. Consistent with the “impaired ciliary gating” mechanism described in the previous section, loss of RPGR function disrupts the glutamylation of tubulin and compromises the diffusion barrier. This leads to the specific failure of opsin trafficking and subsequent rod photoreceptor degeneration [133, 134, 135]. Similarly, *TMEM216*, encoding a transition zone protein classically associated with syndromic Joubert syndrome, was recently identified as a cause of non‐syndromic RP. Mechanistically, specific non‐coding variants in *TMEM216* were found to reduce its expression in the retina, thereby compromising ciliary function without triggering systemic defects [49]. Another X‐linked gene, *RP2*, encodes a protein enriched at the base of the photoreceptor cilium [136]. RP2 facilitates the recruitment of cargo proteins from the endomembrane system to the ciliary base, enabling their entry into the cilia via the IFT machinery [137]. Mutations in *RP2* cause mislocalization of IFT components, such as IFT20, thereby impairing protein trafficking within the photoreceptor cilium [138]. Additionally, *USH2A* is one of the most frequently mutated genes in autosomal recessive RP [139]. The encoded protein, usherin, localizes to the periciliary membrane complex, a specialized microdomain at the apical inner segment that encircles the connecting cilium [140]. Functioning as a molecular tether, usherin anchors the inner segment membrane to the ciliary surface via its extensive extracellular domain. This interaction is critical for maintaining the structural stability of the photoreceptor cilium interface, and its disruption leads to progressive photoreceptor degeneration [141].

The genetic heterogeneity of RP is further exemplified by mutations in genes governing ciliary structural maintenance and cytoskeletal dynamics. *Male Germ Cell‐Associated Kinase* (*MAK*) encodes a cilium‐associated kinase that localizes to the connecting cilium and is essential for regulating ciliary length and axonemal integrity. Its dysfunction leads to destabilized outer segments and progressive degeneration [142]. Similarly, *RP1* encodes a microtubule‐associated protein required for the organization of outer segment discs and axonemal stability. Mutations in *RP1* disrupt the structural scaffold of the cilium, causing both autosomal dominant and recessive RP [143]. Expanding on these structural mechanisms, *TBC1D32* was recently identified as a critical regulator of retinal ciliogenesis. Variants in this gene disrupt the necessary assembly processes of the photoreceptor cilium, leading to broad structural defects and subsequent degeneration [55]. Furthermore, recent studies have highlighted the critical role of actin dynamics at the ciliary base, particularly involving the photoreceptor cilium actin regulator (PCARE, also known as C2orf71 or KIZUNA). The PCARE protein localizes to the base of the photoreceptor connecting cilium, where it recruits the actin‐remodeling factor WASF3 to facilitate the expansion of new outer segment discs. Loss of PCARE disrupts this actin‐regulatory module, resulting in severe ciliary structural defects and representing a prevalent cause of autosomal recessive RP in multiple populations [144].

#### 4.1.2 Leber Congenital Amaurosis (LCA)

LCA is a severe inherited retinal disorder that typically manifests in infancy, characterized by profound visual impairment, nystagmus, and reduced or absent pupillary light reflexes [145]. LCA is a highly heterogeneous disorder, with mutations in over 25 genes (e.g., *RPE65*, *CEP290*, *GUCY2D*) currently identified, which contributes to its variable clinical presentation [2, 146, 147]. As one of the most common genetic causes of congenital blindness in children, the pathogenesis of LCA is closely linked to mutations in various genes that disrupt photoreceptor function, with defects in ciliary genes constituting a major causative mechanism [148].

*CEP290* is the most frequently mutated ciliary gene in LCA [149]. As a core component of the Y‐link structure in the connecting cilium, CEP290 is indispensable for the structural integrity of the transition zone. Defects in this gene exemplify the “gating and trafficking compromise” pathway: mutant photoreceptors fail to assemble a functional transition zone, resulting in the massive mislocalization of inner segment proteins and preventing outer segment biogenesis entirely [24, 150]. Another critical gene, *Crumbs homologue 1* (*CRB1*), encodes the Crumbs homolog 1 protein, which is predominantly expressed in photoreceptor inner segments and Müller glia [151]. This protein is essential for cell polarity, intercellular adhesion, and normal retinal morphogenesis [152]. Mutations in *CRB1* disrupt laminar organization, compromise cell junctions, and cause loss of polarity, resulting in structural disintegration of the retina and progressive photoreceptor death [153].

#### 4.1.3 Cone‐Rod Dystrophy (CRD)

CRD comprises a group of inherited retinal disorders with marked clinical and genetic heterogeneity [154]. The defining pathological feature is progressive degeneration of cone photoreceptors, followed by subsequent rod photoreceptor loss, in a sequence opposite to that observed in RP [155]. Research has revealed an expanding repertoire of causative genes, with a significant fraction encoding ciliary proteins. Mutations in these genes induce photoreceptor death through mechanisms such as disrupted ciliary protein trafficking, impaired phototransduction, or instability of outer segment discs [156].

For example, mutations in the *RPGR* gene represent a significant cause of CRD. Mutations in *RPGR* disrupt normal trafficking of cone opsins and other critical proteins to the membranous discs. These defects result in protein mislocalization and pronounced cone degeneration, which clinically manifests as CRD [157]. Notably, different mutations in the same gene can also lead to classical RP with predominant rod impairment, suggesting considerable overlap in clinical phenotype [158]. In addition, *ABCA4* encodes a retinal‐specific ABC transporter that mediates transport of all‐trans‐retinal aldehyde in the membranous discs of photoreceptors [159]. Given that outer segment discs are specialized structures formed within the modified primary cilium, defects in this disc‐resident protein directly compromise the homeostasis of the ciliary compartment. Consequently, mutations in this gene lead to accumulation of toxic vitamin A derivatives in the retina, impairing both cone and rod photoreceptors and producing a CRD‐like phenotype [160].

### 4.2 Syndromic Inherited Retinal Diseases

#### 4.2.1 Bardet–Biedl Syndrome (BBS)

BBS is a rare autosomal recessive ciliopathy characterized by complex clinical manifestations and high genetic heterogeneity [161]. Core clinical features include retinal dystrophy leading to progressive visual impairment or blindness, early‐onset obesity, postaxial polydactyly, and renal developmental anomalies or functional impairment. Additional manifestations may include intellectual disability, hypogonadism, and metabolic disturbance, representing a broad and variable disease spectrum [162].

The pathogenesis of BBS is directly linked to defects in ciliary structure and function [163]. For instance, the *BBS1* gene encodes a core component of the BBSome, a protein complex essential for cargo recognition and trafficking at the ciliary base [164]. A prevalent mutation in this gene disrupts BBSome assembly, leading to mislocalization of key phototransduction proteins and subsequent impairment of photoreceptor outer segment maintenance. This process ultimately triggers progressive retinal degeneration [165]. Furthermore, *Meckel syndrome type 1* (*MKS1*) encodes a core component of the ciliary transition zone, functioning as a structural scaffold for the assembly of multiple protein complexes [166]. Mutations in this gene compromise the ciliary gate function, permitting abnormal protein influx and disrupting the precise molecular composition of the photoreceptor cilium [167]. The resulting structural and functional abnormalities in photoreceptors represent a key pathogenic mechanism contributing to the retinal manifestations of BBS.

#### 4.2.2 Joubert Syndrome (JS)

JS is a congenital cerebellar ataxia inherited in an autosomal recessive or X‐linked manner, frequently associated with multisystem involvement, including abnormalities of the retina, kidneys, skeleton, and liver [168]. Retinal defects, such as retinal dystrophy or ocular structural anomalies, occur in approximately 80% of patients [169]. To date, numerous causative genes have been identified, most of which encode proteins localized to the ciliary axoneme, basal body, transition zone, or components involved in IFT. Mutations in these genes disrupt ciliary assembly, signaling, or protein trafficking, leading to multiorgan developmental defects [170, 171, 172].

For instance, the *CEP290* mutation, previously discussed as a major cause of LCA, is also a frequent cause of JS. Mutations in this gene impair the integrity of the diffusion barrier, causing abnormal protein composition within the cilia [173]. *AHI1* was the first JS‐associated gene. Its encoded protein is essential for ciliogenesis, and pathogenic mutations disrupt ciliary structure and perturb IFT, ultimately resulting in degeneration of photoreceptor [174].

#### 4.2.3 Usher Syndrome (USH)

USH is a classic autosomal recessive ciliopathy defined by combined sensorineural hearing loss and retinitis pigmentosa, representing the most common cause of concomitant deafness and blindness [175]. USH exhibits marked clinical heterogeneity and is categorized into three major subtypes (USH1–3), which are distinguished by the severity and progression of hearing loss and the age of onset for vestibular dysfunction [176]. Each clinical subtype is associated with specific genetic mutations, allowing further genotype‐based stratification [177].

To date, mutations in 11 distinct genes have been identified as causative for this disorder [178]. For instance, mutations in the *myosin VIIA* (*MYO7A*) gene are responsible for the most severe subtype, USH1B [179]. This gene encodes an unconventional myosin that functions as a molecular motor. In photoreceptors, this protein mediates transport of opsin and other phototransduction components from the inner segment to the outer segment through the connecting cilium [180]. Loss of *MYO7A* impairs this trafficking process, leading to progressive photoreceptor degeneration [181]. In contrast, the *USH2A* gene plays a major role in the most common subtype, USH2A [182]. Its encoded protein, usherin, localizes to the periciliary region surrounding the connecting cilium of the photoreceptor and is also transiently expressed in the developing cochlear hair cells. Defects in usherin compromise the ciliary structural integrity in both sensory organs, leading to the combined phenotype of retinitis pigmentosa and sensorineural hearing loss [140].

## 5 Targeting the Cilium for the Treatment of Inherited Retinal Diseases

Structural or functional abnormalities of the photoreceptor cilium, arising from mutations in ciliary genes, have been identified as a major pathogenic mechanism in IRDs [12]. Consequently, targeting these ciliary genes has emerged as a promising strategy for the intervention of these conditions. Current approaches primarily focus on two major directions: gene therapy and small‐molecule drug therapy [13] (Table 2).

**TABLE 2:** Therapeutic strategies targeting the photoreceptor cilium for inherited retinal diseases.

Therapeutic modality	Strategy/candidate	Target gene/mechanism	Target disease	Current development stage
Gene augmentation	Voretigene neparvovec (Luxturna)	*RPE65*	LCA2	Approved (FDA/EMA)
	AAV‐RPGR	*RPGR*	X‐linked RP	Phase I/II/III
	AAV‐RAB28	*RAB28*	CRD	Preclinical (Zebrafish)
Gene editing	EDIT‐101 (CRISPR/Cas9)	*CEP290*	LCA10	Phase I/II
	Prime editing (eVLPs)	Correction of pathogenic variants (e.g., *RPE65, MFRP*)	RP/LCA	Preclinical
	mRNA‐LNP therapeutics	Transient expression of ciliary proteins (e.g., *USH2A, CEP290*)	USH/RP/LCA	Preclinical
Pharmacological Therapy	HDAC6 inhibitors	Inhibition of Tubulin deacetylation	RP	Preclinical
	Emixustat	Inhibition of RPE65 (Visual cycle modulation)	STGD1	Preclinical
	NTR/Mtz Screening Hits	Novel neuroprotective compounds	RP	Preclinical

### 5.1 Gene Therapy

Gene therapy aims to directly correct pathogenic variants in ciliary genes, thereby restoring the structural integrity and functional capacity of the photoreceptor. The eye is considered an ideal target organ due to its relative immune privilege and anatomical enclosure, which limit systemic dissemination of viral vectors and reduce the risk of systemic immune reactions [14]. This approach is typically administered via subretinal or intravitreal injection to achieve efficient transfection in specific retinal cell types [183].

Gene augmentation therapy using adeno‐associated virus (AAV) vectors represents the most promising strategy. This approach is particularly suitable for recessive IRDs, where delivery of a functional gene copy compensates for loss‐of‐function mutations. The most prominent milestone in this field is the FDA approval of Luxturna (voretigene neparvovec) for the treatment of *RPE65*‐associated LCA2. By delivering a normal *RPE65* gene, this therapy restores the visual cycle within the retinal pigment epithelium, ensuring a continuous supply of visual pigment to the photoreceptor outer segments [184]. Although *RPE65* is not a structural ciliary protein, its clinical success has established a paradigm that paved the way for targeting specific ciliary genes. Building on this momentum, AAV‐based augmentation therapies for X‐linked RP caused by mutations in *RPGR* have demonstrated encouraging efficacy in clinical trials, with several programs advancing to phase III [185, 186]. In parallel with these clinical advances, preclinical research continues to expand the therapeutic landscape. Notably, recent work by Moran et al. utilized a zebrafish model of *RAB28*‐associated cone‐rod dystrophy to demonstrate that cone‐specific gene augmentation successfully restored outer segment phagocytosis functionality [187]. This compelling preclinical evidence validates the therapeutic potential of cell‐type‐specific gene replacement modalities.

For dominant disorders or mutation types where simple gene supplementation is insufficient, genome editing technologies such as CRISPR/Cas9 offer a more precise modality. These tools enable direct correction of pathogenic variants at the DNA level [188]. EDIT‐101, a pioneering in vivo genome‐editing therapy for LCA type 10, targets a recurrent intronic mutation in the *CEP290* gene [189]. This therapeutic strategy employs an AAV5 vector to deliver CRISPR/Cas9 components, which precisely excise the pathogenic intronic sequence, thereby restoring normal splicing and protein function of CEP290 and rectifying the underlying ciliary defect. Phase I/II clinical data have demonstrated preliminary safety and feasibility for this approach [190]. However, addressing the efficiency and delivery limitations of traditional editing, studies in 2024 have developed engineered virus‐like particles (eVLPs) to deliver prime editors, demonstrating therapeutically relevant correction rates for pathogenic variants in retinal models compared to earlier iterations [191].

Despite the widespread use of AAV vectors, limitations such as restricted packaging capacity (< 4.7 kb) and potential immunogenicity have spurred interest in non‐viral delivery systems, particularly lipid nanoparticles (LNPs) [192, 193]. LNPs can efficiently encapsulate CRISPR components and support transient editing activity, potentially reducing risks associated with long‐term nuclease expression. More recently, in 2024, Antas et al. highlighted the emerging potential of mRNA‐based therapeutics delivered via LNPs as a cost‐effective and non‐viral alternative for transiently expressing ciliary proteins, offering a safer profile for treating recessive IRDs [6].

### 5.2 Small‐Molecule Pharmacological Therapy

Small‐molecule drugs provide an interim therapeutic strategy by modulating cilia‐associated pathways or counteracting downstream pathological events. These agents can be administered non‐invasively, such as orally, and generally have lower costs, potentially benefiting a broader patient population while definitive gene therapies are under development [194]. However, the identification of effective lead compounds requires robust and efficient screening platforms. As highlighted in a recent review by Xiao et al., zebrafish models are uniquely positioned to meet this challenge. Their high fecundity permits large‐scale analysis, while their optical transparency enables non‐invasive in vivo imaging of retinal morphology. These features facilitate high‐throughput, phenotype‐based screening [195]. A prime example is the application of the nitroreductase (NTR)/metronidazole (Mtz) system to induce specific photoreceptor ablation. This inducible model has enabled large‐scale in vivo phenotypic screens, leading to the identification of multiple novel neuroprotective compounds. This approach serves as a powerful complementary route for therapeutic discovery [196].

In parallel with these phenotypic discovery efforts, mechanism‐based strategies that directly target ciliary dynamics have also shown promise. A direct pharmacological approach to targeting ciliary dynamics involves histone deacetylase 6 (HDAC6) inhibitors [197]. HDAC6 deacetylates tubulin and other substrates, exerting a negative effect on IFT. Pharmacological inhibition of HDAC6 has been shown to enhance IFT and improve the function of molecular motor proteins. In preclinical models, this strategy has partially restored ciliary trafficking and delayed photoreceptor degeneration [11]. Other small molecules act indirectly by alleviating stressors on the ciliary compartment. For example, emixustat, a visual cycle modulator, reversibly inhibits RPE65 activity [198]. Slowing the visual cycle, it reduces the accumulation of toxic bisretinoids like lipofuscin in the retina [199]. Although these agents do not correct the underlying genetic defects, they may provide temporary symptomatic relief or disease‐modifying effects for patients with specific genetic backgrounds.

## 6 Conclusion and Perspectives

The photoreceptor cilium, with its intricate architecture spanning from the basal body to the axoneme and its highly regulated IFT system, is indispensable for retinal homeostasis and visual function [17]. Accumulating genetic evidence linking a vast number of IRDs to mutations in ciliary genes unequivocally establishes ciliary dysfunction as a central pathogenic mechanism [12, 13]. Despite significant progress in identifying numerous ciliary genes associated with IRDs, various challenges remain. A substantial fraction of IRD cases remain genetically unresolved, underscoring a critical gap in our understanding [200].

To bridge this diagnostic gap, future research must address two distinct layers of genetic complexity. First, at the mechanistic level, a rigorous analysis of genotype‐phenotype correlations is required to explain the extensive phenotypic heterogeneity. A prime example is *PCARE*; despite its ubiquitous expression, pathogenic variants predominantly manifest as non‐syndromic RP due to the specialized demand of photoreceptors for *PCARE*‐dependent actin dynamics [144]. Similarly, mutation class effects (as seen in *CEP290*) and alternative splicing (e.g., *RPGR* ^ORF15^) dictate disease severity and tissue specificity [4, 201]. Second, at the population level, the translational relevance of current research is limited by the lack of ethnic diversity in genomic databases [202]. Founder mutations and population‐specific variant spectra, exemplified by the high prevalence of *EYS* variants in East Asians and the enrichment of homozygous mutations in Arab populations due to consanguinity, are frequently missed by Eurocentric diagnostic panels [5, 203]. Therefore, integrating deep mechanistic studies with multi‐ethnic genomic investigations is imperative for the design of inclusive screening strategies.

In parallel with these genetic insights, therapeutic strategies for IRDs are rapidly evolving beyond conventional viral vector‐based gene therapies toward less invasive and more versatile modalities. A promising frontier is the development of non‐invasive drug delivery systems. While intravitreal injections remain the standard, they carry inherent risks of complications. Recently, Shen et al. pioneered a non‐invasive eye drop formulation based on fluorocarbon‐modified chitosan (FCS) nanocomplexes, which effectively delivered anti‐vascular endothelial growth factor A (VEGFA) antibodies in a mouse model, achieving outcomes comparable to intravitreal injections [204]. This innovation holds transformative potential for IRD management by enabling safe, chronic, at‐home administration, thereby improving patient compliance and reducing treatment burden. In parallel, novel molecular tools are emerging. Targeted protein degradation technologies, such as proteolysis‐targeting chimeras (PROTACs) and lysosome‐targeting chimeras (LYTACs), offer a powerful approach to eliminate toxic gain‐of‐function or dominant‐negative proteins resulting from gene mutations [205]. For instance, a bispecific aptamer‐based LYTAC system has been designed to bind and direct VEGF to lysosomes for degradation, demonstrating the therapeutic potential of this modality in retinal disorders [206]. However, a fundamental challenge lies in achieving selective removal of mutant proteins within the structurally constrained ciliary compartment without disrupting its overall architecture. Moreover, ensuring absolute specificity in degrader design to prevent off‐target effects on wild‐type proteins remains a critical frontier for future development.

A unique perspective emerging from recent studies is the potential role of ciliary signaling pathways in modulating retinal immune responses [207, 208]. Emerging evidence suggests that ciliary dysfunction may trigger sterile inflammation, contributing to photoreceptor degeneration [209]. Targeting these inflammatory pathways, either through small‐molecule inhibitors or immunomodulatory therapies, could provide a complementary strategy to address the secondary consequences of ciliary defects. Additionally, the integration of advanced technologies such as CRISPR‐based gene editing and induced pluripotent stem cell (iPSC)‐derived retinal organoids offers unprecedented opportunities for personalized medicine, enabling the development of patient‐specific therapies tailored to individual genetic profiles [210]. In conclusion, while significant strides have been made in understanding the role of ciliary dysfunction in IRDs, substantial challenges remain. Future research must focus on unraveling the genetic and mechanistic complexities of IRDs, developing innovative therapeutic modalities, and addressing the translational barriers to clinical implementation. By leveraging multidisciplinary approaches, including genetics, molecular biology, and nanotechnology, we can pave the way for transformative therapies that restore vision and improve the quality of life for individuals affected by IRDs.

### 6.1 Methodology

To ensure a comprehensive and rigorous review of ciliary defects in IRDs, we conducted a systematic literature search using PubMed and OMIM databases up to January 2026. Key search terms included “inherited retinal diseases,” “photoreceptor cilium,” “ciliary genes,” and specific disease subtypes (e.g., “Retinitis Pigmentosa,” : “Leber Congenital Amaurosis”). The primary selection of IRD‐associated causative genes was based on authoritative records from the Retinal Information Network (RetNet; https://sph.uth.edu/retnet/). To ensure the reliability of the summarized data, we applied strict inclusion criteria: we prioritized peer‐reviewed articles that provided experimental evidence linking specific gene mutations to ciliary structural or functional defects in photoreceptors. Furthermore, to accurately summarize available animal models, we cross‐referenced the Mouse Genome Informatics (MGI) and Zebrafish Information Network (ZFIN) databases to verify the established vertebrate models listed in this review.

## Author Contributions

G.G. and L.L wrote the manuscript and drew the figures. J.R. and J.Z. supervised the project and edited the manuscript.

## Conflicts of Interest

The authors declare no conflicts of interest.
